# Volatile Profile of Cashew Apple Juice Fibers from Different Production Steps

**DOI:** 10.3390/molecules20069803

**Published:** 2015-05-27

**Authors:** Ana Carolina de Oliveira Nobre, Áfia Suely Santos da Silva de Almeida, Ana Paula Dajtenko Lemos, Hilton César Rodrigues Magalhães, Deborah dos Santos Garruti

**Affiliations:** 1 Post-Graduation Program in Natural Resources, State University of Ceara, Av. Dr. Silas Munguba, 1700, Campus do Itaperi, Fortaleza, CE 60740-000, Brazil; 2 Post-Graduation Program in Food Science and Technology, Federal University of Ceara, Av. Mister Hull, 2977, Block 858, Fortaleza, CE 60356-000, Brazil; E-Mail: afiasuely@yahoo.com.br; 3 Pharmacy Department, Federal University of Ceara, Rua Alexandre Baraúna, 949, Fortaleza, CE 60430-160, Brazil; E-Mail: na_pdajtenko@hotmail.com; 4 Laboratory of Sensory Analysis, Embrapa Tropical Agroindusytry, Av. Dr. Sara Mesquita, 2270, CP 3761, Fortaleza, CE 60511-110, Brazil; E-Mails: hilton.magalhaes@embrapa.br (H.C.R.M.); deborah.garruti@embrapa.br (D.S.G.)

**Keywords:** co-products, aroma, flavor chemistry, headspace, SPME

## Abstract

This study aimed to determine the volatile profile of cashew apple fibers to verify which compounds are still present after successive washings and thus might be responsible for the undesirable remaining cashew-like aroma present in this co-product, which is used to formulate food products like vegetarian burgers and cereal bars. Fibers were obtained from cashew apple juice processing and washed five times in an expeller press. Compounds were analyzed by the headspace solid-phase micro extraction technique (HS-SPME) and gas chromatography-mass spectrometry (GC-MS), using a DB-5 column. Sensory analysis was also performed to compare the intensity of the cashew-like aroma of the fibers with the original juice. Altogether, 80 compounds were detected, being esters and terpenes the major chemical classes. Among the identified substances, 14 were classified as odoriferous in the literature, constituting the matrix used in the Principal Component Analysis (PCA). Odoriferous esters were substantially reduced, but many compounds were extracted by the strength used in the expeller press and remained until the last wash. Among them are the odoriferous compounds ethyl octanoate, γ-dodecalactone, (*E*)-2-decenal, copaene, and caryophyllene that may contribute for the mild but still perceptible cashew apple aroma in the fibers that have been pressed and washed five times. Development of a deodorization process should include reduction of pressing force and stop at the second wash, to save water and energy, thus reducing operational costs and contributing to process sustainability.

## 1. Introduction

Brazilian agribusiness is an ever-growing economic sector. Fruit harvesting has been intensively representing this agricultural sector as a result of increased professionalization, exploitation of larger areas, irrigation and the development of new technologies, with the aim of improving quantitative and qualitative fruit production. At the same time, many companies have been investing in tropical fruit processing, looking for the complete utilization of the product.

Northeastern Brazil has a special predominance in this international agribusiness. In this region, the cashew commodity chain generates some 200 thousand tons of nuts and two million tons of apples. The cashew apple’s industrial use generates fibrous residues (fibers) that are commonly reused to enrich animal feed or discarded, due to the lack of monetary incentive for its use as a human food [1].

The elaboration and consumption of products coming from this cashew apple co-product, a rich source of fiber, vitamin C and carotenoids, could provide utilization alternatives, besides the possibility of diversifying the population’s diet [2], thus presenting a valuable option for the waste of this important raw material [3]. Cashew apple is a fruit with outstanding sensory properties and its processing co-products are produced with the cashew aroma still present. These materials must then be deodorized or not, depending on the intended industrial use.

A range of new cashew-derived products are being developed in research institutions, such as burgers, cakes, pastries, cereal bars, among others [4,5,6]. The proposal of the hamburger made with cashew apple fibers is to elaborate a product similar to the beef hamburger, but yet entirely vegetable, as an option for special consumers, such as vegetarians, for instance. In order to reduce the size and the flavor of the fruit in the hamburger, Lima *et al.* pre-processed the fiber by successive fiber washings with water performed by expeller pressing [7]. However, even after the fiber washing and addition of other ingredients, such as soy or bean protein, the hamburger still presents a cashew-like aroma and flavor. Similarly, other products also present the characteristic fruit flavor, which could be an issue in their commercialization.

It is known that the nutritional richness of a given food, as well as the aspects related to its color, appearance, preservation and presentation impart important characteristics, influencing consumers’ choices and acceptance of such products, therefore, these foods derived from cashew fibers do not yet present the necessary acceptability for a commercial product.

The flavor of foods is an integrated response to taste and aroma sensations. Taste is attributed to the non-volatile compounds perceived by the tongue, whereas aroma is something much more complex, due to the tens or even hundreds of volatile substances, representing various chemical classes with different physicochemical properties [8].

Thus, this work aimed to determine the volatile profile of cashew apple fibers to verify which compounds are still present after successive washings in a process used to obtain a fiber with reduced characteristic cashew flavor, with the goal of making this co-product more suitable for the formulation of food products.

## 2. Results and Discussion

The cashew apple processing involved the extraction of the whole juice, a liquid product resulting from the first pressing, and the obtainment of six fiber portions resulting from their respective pressings. The HS-SPME technique, followed by gas chromatography-mass spectrometry analysis (GC-MS), enabled the detection of a total of 80 volatile compounds in the analyzed samples (Table 1), 37 of which were originally present in the cashew juice. Among the juice compounds, 35 were identified, corresponding to 99.4% of the chromatogram area. It was observed that the majority was composed of esters (13 compounds, corresponding to 80.2% of the area), followed by terpenes (eight compounds, 5.6%), hydrocarbons (five compounds, 1.1%) and alcohols (five compounds, 5.8%), aldehydes (four compounds, 1.9%) and, as the minority, carboxylic acids, which included five compounds, but corresponding to only 5.2% of the chromatogram area.

The cashew juice analyzed in the present work showed a similar profile to those described in the literature, but the number of detected compounds was much smaller, what can be explained by several factors, such as the difference in the raw material, the volatile extraction method and the chromatographic conditions used. By using the enrichment of headspace volatile compounds in Porapak polymer by the suction isolation technique, elution with acetone and separation in a VA-WAX column, Garruti *et al.* [9] detected the presence of 58 volatile compounds in cashew apple juice from clone CCP 76, in which esters were the predominant chemical class, followed by aldehydes, carboxylic acids, alcohols, ketones, hydrocarbons, lactones and terpenes. Studying the volatile compounds from the cashew water phase resulting from the juice concentration and extracted with dichloromethane, Sampaio *et al.* [10], identified 71 compounds, with predominance of esters (27), alcohols (21), carboxylic acids (11), aldehydes (4), ketones (4), lactones (3) and one hydrocarbon. In another work with the volatile compounds from the headspace of concentrated cashew juice, Sampaio *et al.* [11] performed volatiles isolation by suction using the Porapak polymer technique and acetone for desorption. The authors in that case reported 70 compounds, with esters in larger quantity (90% of total mass of volatiles), followed by aldehydes (6%) and alcohols (3%).

**Figure 1:**
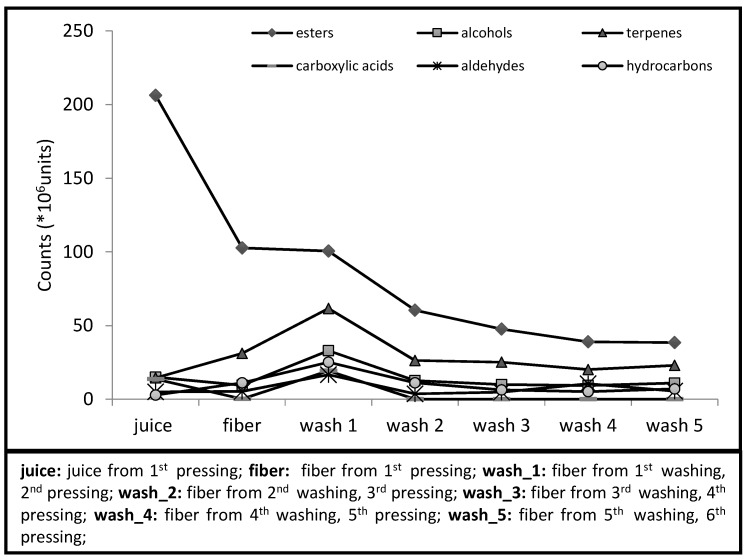
Chromatogram area counts, by chemical class, of headspace volatile compounds of cashew juice and co-products.

Figure 1 shows total area counts of the chromatography peaks by organic function for each product. We can see that the main differences between the volatile profiles of juice and fiber, both obtained in the first pressing, are related to esters and terpenes. The fiber, while still embedded in the juice, presented half of the ester area counts, but a higher content of terpenes. When the fiber underwent another pressing (wash 1), the total amount of esters remained the same, but all the other compounds increased, indicating that they were mechanically released by the pressing force from the matrix, where they were physically or chemically bonded, and therefore, could not be volatilized. The next time the fiber passed through the expeller with water (wash 2) the volatile compounds of all chemical classes were reduced, but further washings had a minor influence on the removal of the volatile fraction.

**Table 1:** Chromatogram area counts of headspace volatile compounds of cashew juice and co-products.

Peak	KI	Compound	Juice	Area Counts × 10^6^					
				Co-Products					
				Fiber	Wash_1	Wash_2	Wash_3	Wash_4	Wash_5
1	<800	3-methylbutanal	0.50 ± 0.0	0.63 ± 0.0	1.02 ± 0.2	nd	nd	nd	nd
2	<800	acetic acid	13.55 ± 2.7	0.07 ± 0.3	13.42 ± 1.9	nd	nd	nd	nd
3	857	ethyl (*E*)-2-butenoate	15.14 ± 0.9	1.28 ± 0.2	nd	nd	nd	nd	nd
4	860	ethyl 2-methyl butanoate	10.62 ± 1.4	1.90 ± 0.2	nd	nd	nd	nd	nd
5	866	ethyl 3-methyl butanoate	130.05 ± 30.7	21.20 ± 4.5	6.77 ± 1.5	1.70 ± 0.3	nd	nd	nd
6	958	ethyl (*E*)-2-methyl-2-butenoate	6.92 ± 1.2	nd	0.73 ± 0.2	nd	nd	nd	nd
7	976	ethyl 3-methyl pentanoate	6.47 ± 1.1	0.41 ± 0.1	2.40 ± 0.4	1.04 ± 0.1	nd	nd	nd
8	981	ethyl 4-methyl pentanoate	1.89 ± 0.3	nd	0.52 ± 0.0	nd	nd	nd	nd
9	1004	ethyl hexanoate	27.33 ± 3.8	15.51 ± 2.0	9.23 ± 1.2	1.92 ± 0.3	1.56 ± 0.2	0.97 ± 0.1	nd
10	1012	octanal	2.65 ± 0.2	nd	0.95 ± 0.0	nd	nd	nd	nd
11	1034	limonene	nd	nd	0.96 ± 0.2	0.41 ± 0.1	nd	nd	nd
12	1057	3-methylbutyl butanoate	1.24 ± 0.1	1.22 ± 0.1	2.03 ± 0.2	0.89 ± 0.1	0.39 ± 0.0	0.31 ± 0.0	0.11 ± 0.0
13	1065	amyl butanoate	nd	nd	0.90 ± 0.2	nd	nd	nd	nd
14	1081	1-octanol	2.95 ± 0.1	nd	2.80 ± 0.2	nd	nd	nd	nd
15	1088	terpinolene	nd	nd	1.15 ± 0.1	nd	nd	nd	nd
16	1102	ethyl heptanoate	nd	0.93 ± 0.1	2.63 ± 0.2	0.81 ± 0.1	0.64 ± 0.0	0.46 ± 0.0	0.39 ± 0.0
17	1111	(*E*)-2-nonen-1-ol	8.97 ± 0.5	8.17 ± 0.4	12.12 ± 0.7	10.57 ± 0.6	9.13 ± 0.5	8.90 ± 0.5	8.77 ± 0.5
18	1152	amyl 3-methyl butanoate	nd	0.23 ± 0.0	0.96 ± 0.0	0.23 ± 0.0	0.19 ± 0.0	nd	nd
19	1181	1-nonanol	2.05 ± 0.3	nd	2.06 ± 0.3	nd	nd	nd	nd
20	1194	ethyl 7-octenoate	nd	0.80 ± 0.2	1.57 ± 0.2	0.60 ± 0.0	0.55 ± 0.0	0.42 ± 0.0	0.40 ± 0.0
21	1200	ethyl octanoate	2.91 ± 0.3	38.86 ± 4.2	31.65 ± 3.5	39.37 ± 4.0	32.42 ± 3.6	25.45 ± 2.8	24.24 ± 2.5
22	1214	decanal	1.39 ± 0.1	0.42 ± 0.0	1.45 ± 0.2	0.68 ± 0.0	0.81 ± 0.0	0.72 ± 0.0	0.99 ± 0.1
23	1244	(*Z*)-3-hexenyl isovalerate	nd	0.20 ± 0.0	4.50 ± 0.5	0.20 ± 0.0	0.16 ± 0.0	nd	nd
24	1248	hexyl 3-methyl butanoate	nd	0.28 ± 0.0	nd	0.37 ± 0.0	0.24 ± 0.0	0.32 ± 0.0	0.32 ± 0.0
25	1254	3-methylbutyl hexanoate	nd	2.63 ± 0.5	6.93 ± 0.9	3.24 ± 0.5	2.47 ± 0.4	2.26 ± 0.4	2.00 ± 0.3
26	1277	2-butyl-1-octanol	nd	nd	0.89 ± 0.1	nd	nd	nd	nd
27	1274	(*E*)-2-decenal	nd	1.17 ± 0.2	5.09 ± 0.7	0.92 ± 0.2	0.81 ± 0.2	1.09 ± 0.2	1.08 ± 0.2
28	1290	pentyl hexanoate	nd	nd	0.90 ± 0.1	0.10 ± 0.0	nd	nd	nd
29	1297	ethyl nonanoate	0.30 ± 0.0	1.78 ± 0.2	2.32 ± 0.2	1.18 ± 0.1	0.96 ± 0.1	0.89 ± 0.1	1.13 ± 0.1
30	1302	tridecane	nd	0.34 ± 0.0	1.04 ± 0.2	0.21 ± 0.0	nd	nd	nd
31	1312	undecanal	nd	nd	0.44 ± 0.0	nd	nd	nd	nd
32	1318	6-methyltridecane	nd	nd	0.39 ± 0.0	nd	nd	nd	nd
33	1345	NI	nd	0.26 ± 0.0	0.39 ± 0.0	nd	nd	nd	nd
34	1357	α-cubebene	9.21 ± 0.2	8.11 ± 0.2	3.04 ± 0.1	2.67 ± 0.1	2.36 ± 0.1	nd	nd
35	1372	2-methyltridecane	nd	0.61 ± 0.0	nd	nd	0.31 ± 0.0	0.24 ± 0.0	0.60 ± 0.0
36	1377	2,6,10-trimethyldodecane	nd	nd	4.69 ± 0.5	0.87 ± 0.1	0.39 ± 0.0	0.22 ± 0.0	0.83 ± 0.1
37	1382	NI	0.67 ± 0.0	1.33 ± 0.0	1.89 ± 0.1	0.57 ± 0.0	0.52 ± 0.0	nd	nd
38	1383	octyl butanoate	nd	0.99 ± 0.0	nd	nd	nd	0.42 ± 0.0	0.70 ± 0.0
39	1391	copaene	0.28 ± 0.0	3.63 ± 0.5	4.11 ± 0.5	2.42 ± 0.4	2.48 ± 0.4	1.73 ± 0.2	2.22 ± 0.3
40	1398	ethyl decanoate	0.50 ± 0.1	6.06 ± 0.3	7.52 ± 0.3	4.97 ± 0.2	4.37 ± 0.1	3.73 ± 0.1	4.03 ± 0.1
41	1402	tetradecane	0.27 ± 0.0	6.35 ± 0.3	11.41 ± 0.8	5.23 ± 0.2	3.55 ± 0.2	2.93 ± 0.2	4.13 ± 0.2
42	1412	NI	nd	nd	2.97 ± 0.1	nd	nd	nd	nd
43	1416	NI	nd	nd	2.00 ± 0.1	nd	nd	nd	nd
44	1424	cedrene	nd	3.22 ± 0.2	2.22 ± 0.2	1.53 ± 0.1	1.46 ± 0.1	1.00 ± 0.1	1.95 ± 0.1
45	1433	α-santalene	nd	0.60 ± 0.0	1.73 ± 0.3	nd	nd	nd	nd
46	1439	caryophyllene	0.56 ± 0.1	2.81 ± 0.5	2.67 ± 0.4	1.70 ± 0.3	1.55 ± 0.3	1.07 ± 0.2	1.67 ± 0.2
47	1445	(*E*)-α-bergamotene	0.36 ± 0.0	2.54 ± 0.3	3.35 ± 0.4	1.29 ± 0.2	1.11 ± 0.1	0.75 ± 0.0	1.19 ± 0.2
48	1451	3-methylbutyl octanoate	nd	1.50 ± 0.2	2.74 ± 0.2	0.90 ± 0.1	0.86 ± 0.1	0.73 ± 0.0	0.92 ± 0.1
49	1454	(*Z*)-geranylacetone	nd	nd	2.19 ± 0.3	0.95 ± 0.2	0.92 ± 0.2	0.76 ± 0.0	0.98 ± 0.1
50	1461	NI	1.04 ± 0.2	2.74 ± 0.5	4.43 ± 0.6	nd	nd	nd	nd
51	1467	2-methyltetradecane	0.36 ± 0.0	0.69 ± 0.0	1.87 ± 0.1	0.69 ± 0.0	0.48 ± 0.0	nd	0.42 ± 0.0
52	1475	2,6-di-*tert*-butylbenzoquinone	0.48 ± 0.1	1.11 ± 0.1	5.82 ± 0.4	0.61 ± 0.1	0.47 ± 0.0	0.69 ± 0.0	0.65 ± 0.0
53	1484	(*E*)-ethyl cinnamate	1.74 ± 0.3	1.84 ± 0.3	16.99 ± 2.6	1.71 ± 0.2	1.60 ± 0.2	1.43 ± 0.2	1.56 ± 0.2
54	1491	selinene	nd	2.20 ± 0.2	6.06 ± 0.5	1.01 ± 0.1	0.99 ± 0.1	0.88 ± 0.1	0.98 ± 0.1
55	1501	γ-muurolene	nd	6.89 ± 0.4	12.78 ± 1.8	5.71 ± 0.6	5.46 ± 0.6	4.69 ± 0.5	5.57 ± 0.7
56	1506	pentadecane	0.85 ± 0.2	nd	1.63 ± 0.3	nd	nd	nd	nd
57	1513	β-germacrene	nd	0.71 ± 0.0	5.60 ± 0.2	2.77 ± 0.1	2.68 ± 0.1	2.52 ± 0.1	2.86 ± 0.1
58	1517	γ-elemene	nd	2.41 ± 0.3	nd	nd	nd	nd	nd
59	1522	β-bisabolene	1.06 ± 0.3	1.36 ± 0.2	0.81 ± 0.1	nd	nd	nd	nd
60	1534	δ-cadinene	0.42 ± 0.0	3.40 ± 0.3	3.71 ± 0.3	1.84 ± 0.2	1.77 ± 0.2	1.65 ± 0.1	1.81 ± 0.2
61	1551	cadine-1,4-diene	nd	0.61 ± 0.0	0.33 ± 0.0	nd	nd	nd	nd
62	1562	dodecanoic acid	nd	nd	1.26 ± 0.2	nd	nd	nd	nd
63	1574	NI	nd	0.72 ± 0.1	1.15 ± 0.1	0.42 ± 0.0	0.51 ± 0.0	0.43 ± 0.0	0.49 ± 0.0
64	1594	ethyl dodecanoate	nd	1.05 ± 0.2	2.18 ± 0.2	0.94 ± 0.1	0.76 ± 0.1	1.01 ± 0.1	0.85 ± 0.1
65	1603	hexadecane	0.36 ± 0.0	1.79 ± 0.2	1.12 ± 0.1	2.66 ± 0.2	1.05 ± 0.1	1.26 ± 0.01	1.00 ± 0.0
66	1618	tetradecanal	0.44 ± 0.1	0.49 ± 0.0	0.69 ± 0.0	0.54 ± 0.0	0.46 ± 0.0	0.67 ± 0.0	0.95 ± 0.1
67	1645	2,6,10-trimethylpentadecane	0.92 ± 0.2	0.39 ± 0.0	1.59 ± 0.2	0.32 ± 0.0	nd	nd	nd
68	1680	tetradecanol	0.32 ± 0.0	nd	4.13 ± 0.2	0.45 ± 0.0	0.28 ± 0.0	0.47 ± 0.0	0.45 ± 0.0
69	1693	γ-dodecalactone	2.09 ± 0.6	2.87 ± 0.3	4.70 ± 0.3	3.31 ± 0.2	3.84 ± 0.4	4.40 ± 0.5	3.13 ± 0.2
70	1701	heptadecane	nd	0.80 ± 0.1	0.84 ± 0.1	0.77 ± 0.0	0.58 ± 0.0	0.56 ± 0.0	nd
71	1720	pentadecanal	nd	0.89 ± 0.1	2.87 ± 0.3	0.79 ± 0.0	1.37 ± 0.1	1.15 ± 0.1	1.57 ± 0.1
72	1758	tetradecanoic acid	nd	nd	3.83 ± 0.5	0.54 ± 0.0	nd	nd	nd
73	1765	NI	0.47 ± 0.1	1.97 ± 0.5	3.34 ± 0.7	1.17 ± 0.4	1.07 ± 0.2	1.12 ± 0.2	1.31 ± 0.2
74	1801	octadecane	nd	0.14 ± 0.0	0.46 ± 0.0	0.41 ± 0.0	nd	nd	nd
75	1822	hexadecanal	nd	1.75 ± 0.2	2.76 ± 0.2	0.77 ± 0.0	0.69 ± 0.0	0.61 ± 0.0	0.83 ± 0.1
76	1828	pentadecanoic acid	nd	nd	0.34 ± 0.0	nd	nd	nd	nd
77	1857	NI	nd	nd	1.39 ± 0.2	nd	nd	nd	nd
78	1884	1-hexadecanol	nd	nd	4.10 ± 0.2	0.98 ± 0.0	nd	nd	1.70 ± 0.1
79	1901	NI	nd	nd	1.29 ± 0.1	nd	0.63 ± 0.0	6.26 ± 0.3	nd
80	1938	9-hexadecenoic acid	nd	nd	2.87 ± 0.2	nd	nd	nd	nd

KI: Kovats Indices in a DB-5 column; nd: not detected; NI: not identified; Wash: washing.

In Table 1, the behavior of individual compounds can be followed. We can observe that most of the more volatile esters (from the beginning of the chromatogram) were no longer detected in the first and second washings: ethyl (*E*)-2-butenoate (ethyl crotonate, peak 3), ethyl 2-methyl butanoate (peak 4), ethyl (*E*)-2-methyl-2-butenoate (ethyl tiglate, peak 6) and ethyl isohexanoate (peak 8). Ethyl 3-methyl butanoate (ethyl isovalerate, peak 5) and ethyl 3-methylpentanoate (peak 7) were lost only in the third washing, whereas ethyl hexanoate (peak 9) and 3-methylbutyl butanoate (isoamyl butanoate, peak 12) decreased down to the last washing. Ethyl octanoate (peak 21) remained until the last washing, increasing its juice area for fiber and decreasing thereafter. 

On the other hand, many esters were not detected in the juice, but only in the fibers, or increased in quantity with the extractions carried out by the pressings: amyl butanoate (peak 13), ethyl heptanoate (16), amyl 3-methyl butanoate (18), ethyl 7-octanoate (20), Z-3-hexenyl isovalerate (23), hexyl isovalerate (24), isoamyl hexanoate (25), amyl hexanoate (28), octyl butanoate (38), isoamyl octanoate (48), ethyl dodecanoate (64), ethyl nonanoate (29), ethyl decanoate (40) and ethyl cinnamate (53).

A similar behavior was observed for terpenes, since limonene (peak 11), terpinolene (15), cedrene (44), α-santalene (45), selinene (54), γ-muurolene (55), β-germacrene (57), cadine-1,4-diene (61) and γ-elemene (peak 58) were not detected in the juice. The terpenes copaene (39) and caryophyllene (46)—both odoriferous in cashew juice—bergamotene (47) and cadinene (60) increased up to the first and second washings and then decreased with the following washings, just like most of aldehydes and alcohols. Regarding the hydrocarbons, only tridecane, pentadecane, hexadecane and 2,6,10-trimethyl-pentadecane were detected in the juice, the others were incorporated in the fibers after washings, coming from the cashew apple wax coating.

The minority organic compounds were the carboxylic acids. Among these compounds we detected acetic acid. This compound is derived from an incipient fermentation of raw material and is not a part of the volatile profile of the fruit in its natural state, but with its unpleasant aroma it has a strong odoriferous impact on the juice [10]. However, as shown in Table 1, the acetic acid was found only until the first washing (second pressing), but not in the co-products of the subsequent pressings, indicating that the successive washings were effective in removing it, and that the final product is free of this undesirable odor.

Some volatiles present in the juice were still detected in the fiber resulting from the last washing: 3-methylbutyl butanoate (peak 12), (*E*)-2-nonen-1-ol (17), ethyl octanoate (21), decanal (22), ethyl nonanoate (29), copaene (39), ethyl decanoate (40), tetradecane (41), caryophyllene (46), (*E*)-α-bergamotene (47), 2-methyltetradecane (51), 2,6-di-*tert*-butylbenzoquinone (52), (*E*)-ethyl cinnamate (53), δ-cadinene (60), hexadecane (65), tetradecanal (66), tetradecanol (68), γ-dodecalactone (69) and a non-identified ester (peak 73). Ethyl octanoate became a major compound in the fiber from the fifth washing, and terpenes, still detected in smaller amounts, have extremely low thresholds, *i.e.*, present high odoriferous impact.

**Figure 2:**
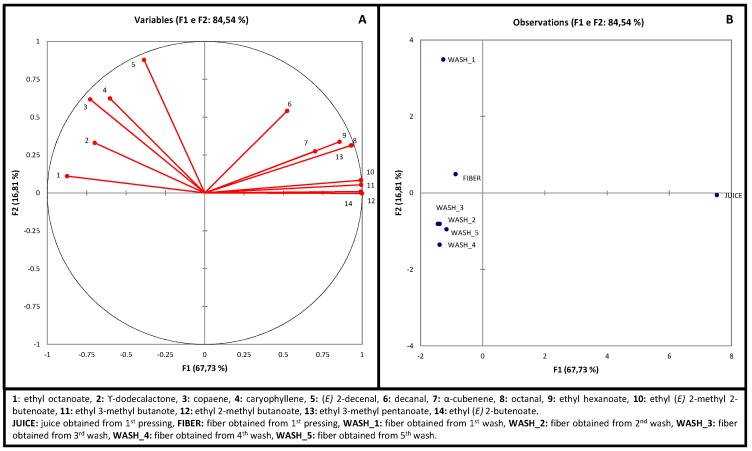
Principal Component Analysis of headspace odoriferous volatile compounds of cashew juice and co-products. (**A**) variables chart (volatile compounds); (**B**) observations chart (samples).

The data obtained from the volatile composition of cashew juice and the co-products were analyzed by Principal Component Analysis (PCA), to determine the compounds’ contribution in discriminating the samples (Figure 2). A selection of variables became necessary in order to make this exploratory analysis more effective. The final matrix was supported by the chromatography peak area of 14 volatile compounds that were selected for having been determined by olfactometric analysis in literature as being odoriferous compounds of medium and high intensity and, therefore, important to the formation of the characteristic cashew aroma and flavor: ethyl esters from the beginning of the chromatogram, particularly ethyl (*E*)-2-butenoate, ethyl 2-methylbutanoate, ethyl 3-methylbutanoate, ethyl 2-methyl (*E*)-2-butenoate, ethyl 3-methylpentanoate and ethyl hexanoate, as well as ethyl octanoate, all associated to the descriptors “cashew”, “fruity”, “sweet” and “floral” [9,10,12,13,14]; aldehydes octanal (green, citric), decanal (pungent, sweet) [9,13] and (*E*)-2-decenal (floral) [15]; the terpenes α-cubebene, copaene and caryophyllene (cashew, sweet, floral) [12,13]; and an important lactone γ-dodecalactone described as “cashew”, “sweet” and “fruity” [10,15].

Figure 2A shows the chart of variables (volatiles compounds) in the first two components. All together, the components F1 and F2 explained 84.54% of the variation among the studied products. Each volatile compound is represented by a vector, whose direction indicates the variable’s growth region and the length of the decomposition on the axes indicates the variable’s importance to differentiate the samples. Figure 2B constitutes the observations chart, in which samples that are located in the same region present similar volatile profiles to one another. PCA discriminated four groups of samples: one group formed only by the cashew juice; another group formed by the original fiber (obtained by the juice extraction); a third group formed by the fiber washed one time and pressed two times (Wash_1); and the last group formed by fibers from the second, third, fourth and fifth washings (Wash_2, Wash_3, Wash_4 and Wash_5, respectively). We observed that component F1 has separated the juice from the fibers, whereas F2 has separated the fibers among them.

By superposing Figure 2A,B, we verify that samples are located near the vectors (variables) that characterize them. The juice showed a rich profile of odoriferous volatile compounds, with a high content of decanal (vector 6), α-cubebene (vector 7), octanal (vector 8) and esters that are represented by vectors 9 to 14 that present sweet, fruity and cashew aromas in the olfactometric studies already cited. In its turn, with the pressing and washings, the fibers reduced or lost most these odoriferous compounds, but presented ethyl octanoate (peak 21), γ-dodecalactone (peak 69), (*E*)-2-decenal (peak 27), copaene (peak 39) and caryophyllene (peak 46) in larger quantities than those found in the juice, which remained in the fibers until the last washing (Table 1). In Figure 2B, we can also observe that Wash_1 is more to the up and left than the original fiber, toward those compounds that are more abundant in the upper quadrant region, corroborating with what has been discussed in Table 1, that Wash_1 presented higher contents of many volatile compounds than the original fiber due to the pressing force that released them from the matrix. Fibers from the second, third, fourth, and fifth washings were located in the bottom left quadrant, presenting poorer profile than the original fiber, by similar among them, indicating that the second washing was able to reduced or even remove these odoriferous compounds, but further washings were no more effective. 

Sensory analysis corroborated with those findings. Table 2 shows the mean values obtained by the sensory panel. We can see that one washing was not enough to reduce the fibers’ cashew-like aroma. With two washings, the aroma decreased significantly, but did not fade away with successive washings. This way, the technological process of washing the cashew fiber should go until the second wash only, saving water, energy, time and costs. 

We also may infer that the five odor active compounds that can still be detected in the last washings in considerable amounts (ethyl octanoate, γ-dodecalactone, (*E*)-2-decenal, copaene, caryophyllene) may have great importance in the characteristic cashew apple flavor. 

**Table 2:** Sensory panel mean of the cashew-like aroma in the fibrous residues.

	Fiber	Wash_1	Wash_2	Wash_3	Wash_4	Wash_5
**Panel Mean**	4.68 ^a^	4.22 ^a^	2.96 ^b^	2.54 ^b^	2.18 ^b^	2.46 ^b^

Means with same letters do not differ significantly (α = 0.05) by REGWq test.

## 3. Experimental Section

### 3.1. Raw Material

Cashew apple samples, composed of a mixture of several clones, were harvested at Embrapa Tropical Agroindustry’s Experimental Field in Pacajus, Ceara State, Brazil, and transported in appropriate boxes to the Laboratory of Agribusiness Processes, where they were stored in a cold chamber (−18 °C) until the following morning for later processing.

### 3.2. Cashew Apple Processing

**Figure 3:**
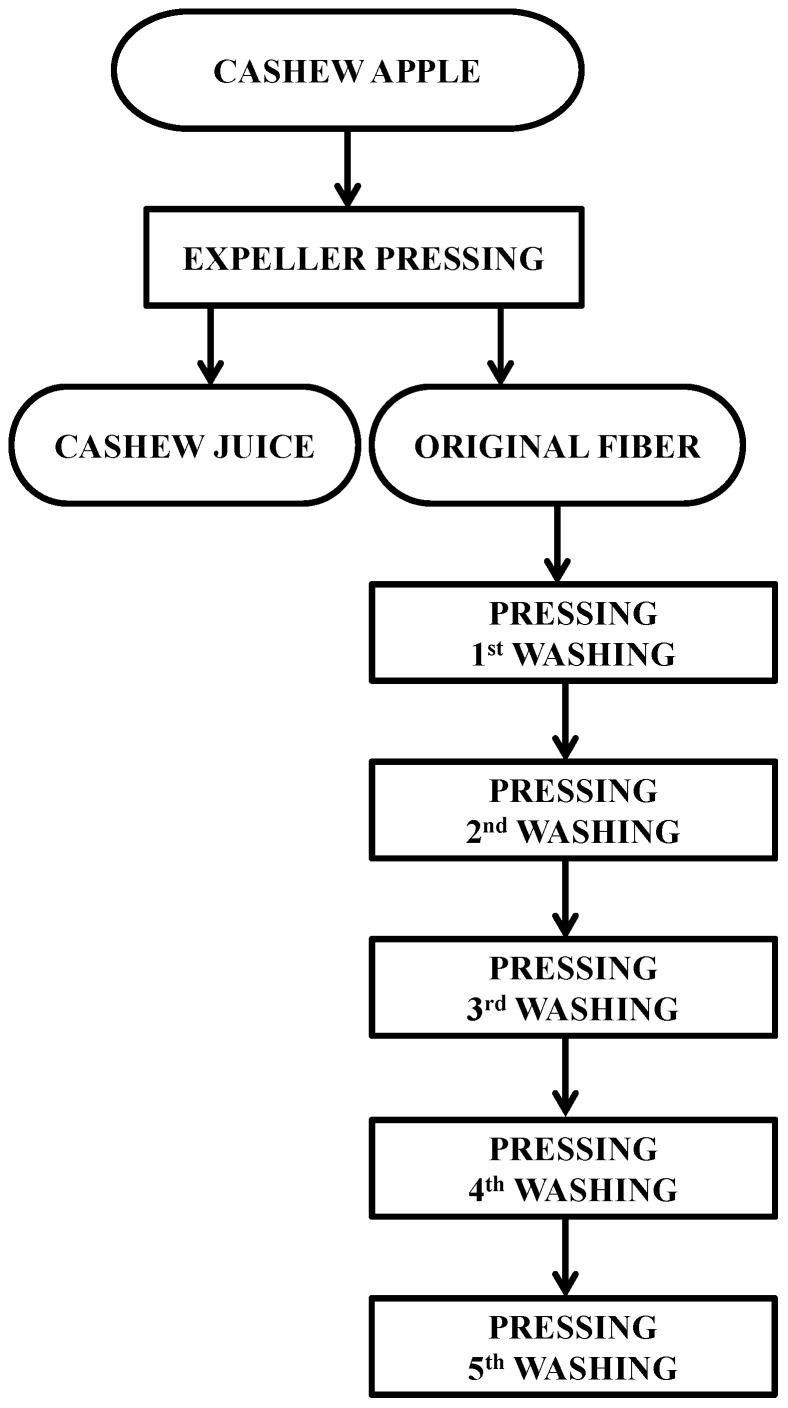
Flowchart of cashew fiber obtainment and treatment.

In order to obtain and treat the fiber, cashew apples were processed as shown by the flowchart in Figure 3. Apples were sanitized by immersion during 30 min in a tank with 80 mL of 10% Cl_2_, dissolved in 80 L of water, and then washed under flowing water. Initially, 30.5 kg were processed by expeller pressing, obtaining cashew juice and the original fiber (fiber from the first pressing). After that, five successive washings in the expeller press were carried out with the addition of water in the same amount by weight as the fibers obtained in the prior pressing. Processing was performed through the continuous stretching of a 21.07 cm long coil, which yielded, at the end of the final pressing, a total of 600 g of cashew fibers. Samples of each step were frozen (‒20 °C) in plastic bags for further volatile compounds analyses.

### 3.3. Isolation of Volatile Compounds

The volatile compounds were isolated from the matrix (juice and fibers) by headspace solid-phase micro extraction (HS-SPME), using a PDMS (100 µm film thickness and 1 cm long) fiber (Supelco, Bellefonte, PA, USA). Cashew juice (5 g) and cashew fibers (2 g) were weighed into 20 mL glass vials with polytetrafluoroethylene/silicone septa. In each sample NaCl 30% (*w*/*v*) was added to reduce the solubility of organic compounds and increase the volatile extraction. Samples were heated at 30 °C in a water bath. The equilibrium time was 5 min, under magnetic stirring at 250 rpm. The SPME fiber was previously conditioned according to the manufacturer’s instructions and then exposed in the headspace for 30 min.

### 3.4. Analysis of Volatiles by Gas Chromatography-Mass Spectrometry (GC-MS)

After the volatiles extraction, the SPME fiber was placed into the injector of a gas chromatograph for thermal desorption of compounds, using the splitlessmode of 01 minute at 200 °C. A Shimadzu GC-2010 (Kyoto, Japan), equipped with mass spectrometry detector (Shimadzu QP-2010) and a DB-5MS (J&W, Agilent Technologies, Santa Clara, CA, USA, 30 m × 0.25 mm × 0.25 µm film width) column was used for the separation and identification. Helium was used as the carrier gas, at 1.5 mL/min flow (column pressure: 13 psi). The initial temperature was 35 °C, remaining for 5 min; then raised to 60 °C, at 4 °C/min, and subsequently to 200 °C, at 15 °C/min, remaining the same for 5 more minutes. Analyses were performed in duplicates for each sample.

### 3.5. Identification of Volatiles

Identification of volatiles was performed by the use of a quadruple mass analyzer at an ionization voltage of 70 eV, based on the comparison mass spectra of retention indices with those of reference compounds, using literature data and database from the National Institute of Standards and Technology [16]. Retention indices (Kovats) were calculated by a series of homologous alkanes (C_8_ to C_24_).

### 3.6. Sensory Analysis

The intensity of the cashew aroma of each one of the six co-products was compared to the cashew juice aroma (reference sample), by means of a structured scale of 9 cm, being 0 = no perceived intensity and 9 = intensity equal to the reference. The analysis was carried out in triplicates by thirteen previously selected judges. The samples were placed in lidded glass cups codified with random 3-digit numbers. The order of presentation was balanced for six samples [17], however, samples were tested in two sessions, along with the reference (cashew juice). Each judge was asked to first smell the aroma from the cup identified as reference and then the other samples.

### 3.7. Statistical Analysis

Sensory data were submitted to ANOVA by the GLM procedure and REGWq test (α = 0.05) for comparison of means using SAS^®^ Statistical Analytical Systems [18] for Windows. Select volatiles data were analyzed by Principal Component Analysis using the XLSTAT software (Version 1.02).

## 4. Conclusions

This study showed that the fibers resulting from the processing of cashew apple juice presented a rich fraction of volatile compounds, even after successive washings. Odoriferous esters were substantially reduced, but many compounds were extracted by the strength used in the expeller press and were present until the last wash. Among them are odoriferous compounds like ethyl octanoate, γ-dodecalactone, (*E*)-2-decenal, copaene, and caryophyllene that may contribute to the mild but perceptible cashew apple aroma in the fibers that have been pressed and washed five times. Development of deodorization process should therefore include reduction of pressing force.

Due to the similarity of volatile profiles and intensity of cashew-like aroma presented among the fibers from the second to the fifth washings, the pre-processing could be finalized at the second wash, to reduce operational costs, with saving of water, energy and time, besides ensuring higher productivity.

This work has contributed to a better understanding of which compounds play a significant role in the formation of cashew apples’ characteristic flavor, aiding future scientific projects focused on the deodorization of this co-product, based on reducing the pressing force during the washing process.
